# Early Prediction of Clinical Response to Etanercept Treatment in Juvenile Idiopathic Arthritis Using Machine Learning

**DOI:** 10.3389/fphar.2020.01164

**Published:** 2020-07-31

**Authors:** Xiaolan Mo, Xiujuan Chen, Chifong Ieong, Song Zhang, Huiyi Li, Jiali Li, Guohao Lin, Guangchao Sun, Fan He, Yanling He, Ying Xie, Ping Zeng, Yilu Chen, Huiying Liang, Huasong Zeng

**Affiliations:** ^1^ Department of Pharmacy, Guangzhou Women and Children’s Medical Center, Guangzhou Medical University, Guangzhou, China; ^2^ School of Pharmaceutical Sciences, Institute of Clinical Pharmacology, Sun Yat-sen University, Guangzhou, China; ^3^ Guangzhou Women and Children’s Medical Center, Institute of Pediatrics, Guangzhou Medical University, Guangzhou, China; ^4^ Guangzhou Women and Children’s Medical Center, Pediatric Allergy Immunology & Rheumatology Department, Guangzhou Medical University, Guangzhou, China; ^5^ Department of Pharmacy, Guangzhou Institute of Dermatology, Guangzhou, China; ^6^ Department of Pharmacy, Sun Yat-sen Memorial Hospital, Sun Yat-sen University, Guangzhou, China

**Keywords:** etanercept, juvenile idiopathic arthritis, machine learning, prediction models, clinical response

## Abstract

**Background and Aims:**

At present, there is a lack of simple and reliable model for early prediction of the efficacy of etanercept in the treatment of juvenile idiopathic arthritis (JIA). This study aimed to generate and validate prediction models of etanercept efficacy in patients with JIA before administration using machine learning algorithms based on electronic medical record (EMR).

**Materials and Methods:**

EMR data of 87 JIA patients treated with etanercept between January 2011 and December 2018 were collected retrospectively. The response of etanercept was evaluated by using DAS44/ESR-3 simplified standard. The stepwise forward and backward method based on information gain was applied to select features. Five machine learning algorithms, including Extreme Gradient Boosting (XGBoost), Random Forest (RF), Gradient Boosting Decision Tree (GBDT), Extremely Random Trees (ET) and Logistic Regression (LR) were used for model generation and validation with fifty-fold stratified cross-validation. EMR data of additional 14 patients were collected for external validation of the model.

**Results:**

Tender joint count (TJC), Time interval, Lymphocyte percentage (LYM), and Weight were screened out and included in the final model. The model generated by the XGBoost algorithm based on the above 4 features had the best predictive performance: sensitivity 75%, specificity 66.67%, accuracy 72.22%, AUC 79.17%, respectively.

**Conclusion:**

A pre-administration model with good prediction performance for etanercept response in JIA was developed using advanced machine learning algorithms. Clinicians and pharmacists can use this simple and accurate model to predict etanercept response of JIA early and avoid treatment failure or adverse effects.

## Introduction

Etanercept is the first and most important tumor necrosis factor (TNF) inhibitor in the treatment of MTX-resistant juvenile idiopathic arthritis (JIA). However, the efficacy of etanercept varies widely among individuals, with only two-thirds of patients responding to it (Otten et al., 2011). Besides, like other biological agents, etanercept can early modify immune components after administration, but it still takes several months to obtain clinical efficacy. During this period, clinicians are unable to revise the main therapeutic regimen, which may delay treatment, resulting in disease progression and substantial economic burden. Furthermore, the adverse effects such as infection and soreness at the injection site should be paid attention to as well (Horneff et al., 2004). Therefore, it is important to predict the efficacy of etanercept before administration and choose an appropriate regimen, which is beneficial to interfere with JIA progression, improve prognosis and reduce the economic burden of patients. Hence, establishing a pre-administration efficacy prediction model of etanercept in JIA is very necessary.

Although etanercept was approved by the FDA for the treatment of JIA in 1999, there were only several studies focused on models for predicting etanercept response in JIA (Otten et al., 2011; Solari et al., 2013; Geikowski et al., 2014; Kearsley-Fleet et al., 2016). Variables excavated by the above models included age onset, the time from disease onset to initiation of etanercept treatment, disease assessment by parents or clinicians. However, these models had certain limitations. First, the existing models were generated using traditional logistic regression method, which may not be the optimal method for model generation. On the other hand, the predictive performance of these models could not be fully evaluated since they had neither validation process nor enough evaluation index such as the area under the curve (AUC). Furthermore, the consequence of these models, which only established based on the European population, may differ from race and region. Due to these conditions, the existing models cannot be widely applied to predict the efficacy of etanercept in JIA accurately.

Therefore, an accurate and widely applicable model is needed to predict the efficacy of etanercept in JIA. Thanks to the powerful data mining and computing capacity of machine learning, it has been widely used in the medical field in recent years. Many advances have been made in medical prediction, such as assistant diagnoses, prognosis evaluation, and new drug development. For example, Nieuwenhove et al. identified an immunological pattern associated with JIA subtypes using machine learning (Van Nieuwenhove et al., 2019); Motwani et al. (2017) used machine learning to predict 5-year mortality in coronary artery disease patients. On the other hand, the increasing number of electronic medical record (EMR) data containing rich comprehensive information of patients such as examination and diagnosis, coupled with the development of machine learning, provides new opportunities for high-performance efficacy prediction model generation (Rahimian et al., 2018). For etanercept, Liu et al. (2019) constructed a model and found that IgG galactosylation status and *MYOM2* gene polymorphism could predict the response of etanercept in ankylosing spondylitis. Moreover, in the model reported by Lewis, et al., the quantification of systemic inflammatory-proteins excavated could predict the long-term treatment response to etanercept in psoriasis (Tomalin et al., 2019). Nonetheless, there hasn’t been any efficacy prediction model of etanercept in JIA generated by machine learning so far.

Therefore, the purpose of this study is to use machine learning to develop an easy-to-use and efficient prediction model based on EMR, to predict the treatment response to etanercept in JIA.

## Methods

### Study Design and Population

This study retrospectively collected the EMR data of children with JIA who were treated at Guangzhou Women and Children’s Medical Center from January 2011 to December 2018. Inclusion criteria were:(1) the diagnosis of patients met the International League of Associations for Rheumatology criteria for JIA (Petty et al., 2004; Martini et al., 2019). (2) the age of onset is 1–16 years old. (3) patients treated with etanercept for at least 3 months and never received any other biological agents before. (4) co-treatment with low-dose corticosteroids and(or) non-steroidal anti-inflammatory drugs and methotrexate were allowed. Exclusion criteria were: (1) combined therapy with other interfering drugs (e.g. biological agents) 3 months before or within the onset of etanercept. (2) etanercept therapy did not reach 3 months. (3) patients with poor compliance with treatment. (4) serious missing of all kinds of medical records. A total of 137 JIA children using etanercept were screened out, but 87 patients were eventually included for the model generation and testing. Then they were randomly assigned into a training set and a test set according to the ratio of 8:2. Besides, we finally collected 14 patients from January 2019 to December 2019 for external validation of the model.

This study was reviewed and approved by the ethics committee of this center (no. 2016021645) and conducted according to the Helsinki declaration. This study was also enrolled in the clinical trial (NCT81603203). Informed consent wasn’t required because the study was retrospective. Data used in this study were anonymous and no identifiable personal data of the patients were available for the analysis.

### Assessment of Etanercept Clinical Response

All patients were treated with etanercept once a week at a dose of 0.8mg/kg. Because of the retrospective study, it is hard to collect subjective indicators such as patients’ and doctors’ global assessment of the disease. Hence, we used DAS44/ESR-3, which is a simplified standard related to the European League of Associations for Rheumatology criteria (Ranganath et al., 2007; Consolaro et al., 2009), to evaluate the efficacy of etanercept instead of JADAS or ACRpedi scoring tools (Giannini et al., 1997; Consolaro et al., 2009). The simplified calculation formula of the disease activity is as follows: $\text{y}=0.53938\sqrt{\text{RAI}}+0.06465*\text{SJC}44+0.33\text{ ln}(\text{ESR})+0.224$ (RAI, Ritchie articular index; SJC, swollen joint count; ESR, erythrocyte sedimentation rate). A significant change of DAS44 score from baseline to 3 months after the start of etanercept was used to define the response. Patients with a significant decrease in DAS44 (>0.6) represented responders, while a decrease of ≤ 0.6 were non-responders.

### Clinical Variables

**Figure 1:**
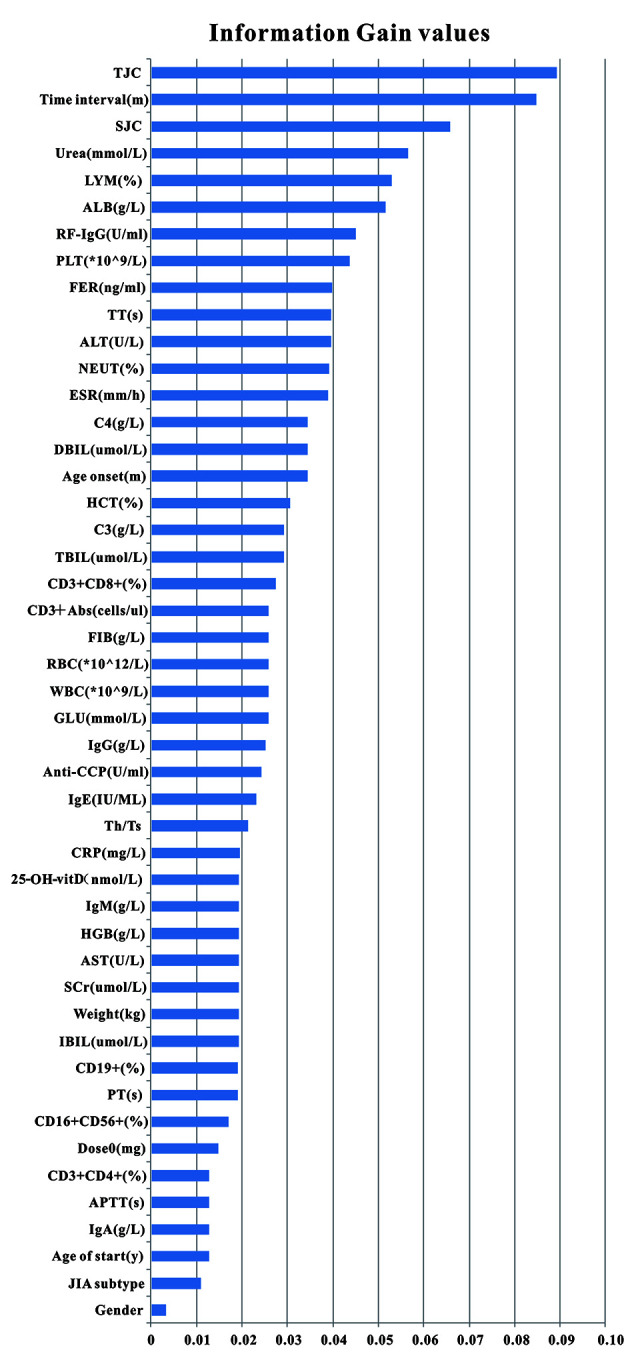
Clinical variables used for model generation and their information gain values. Total 47 pre-administration clinical variables were used to generate models. Variables were ranked according to their information gain values which reflect the entropy gain with respect to the outcome. The longer the blue transverse column (the higher the value), the greater importance on the outcome.

Clinical variables collected in this study were derived from pre-administration EMR. We collected 47 clinical variables, including demographic data (weight, gender, etc.), the acute phase of inflammatory products (C-reactive protein, ESR, etc.), joint conditions (tender joint count, joint imaging, etc.), immune-related indicators (rheumatoid factor, antinuclear antibodies, etc.), liver function (alanine aminotransferase, aspartate aminotransferase, total bilirubin, etc.), renal function, blood routine examination, blood coagulation function (active partial thrombin time, fibrinogen, etc.), related lymphocytes (CD3+abs, CD3+CD4+, etc.), 25-hydroxy-vitamin D, etc. All variables used in feature selection are shown in **Figure 1**, and the full names and abbreviation of variables are shown in **Table 1** in supplementary.

### Machine Learning

**Figure 2:**
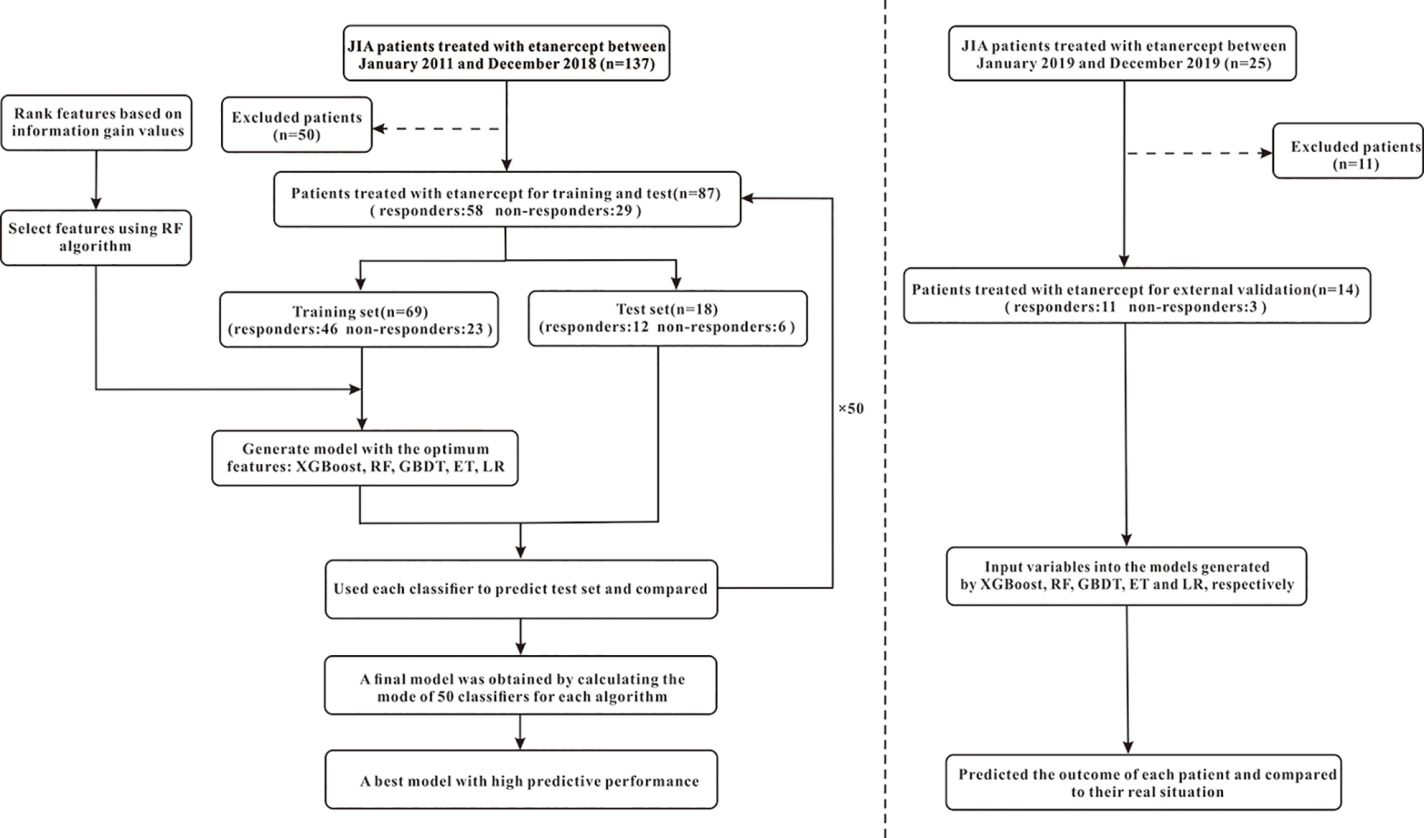
The flowchart of model generation and validation. The left side of the dotted line is the process of model generation, while the right side is the external validation of the model. XGBoost, extreme gradient boosting; RF, random forest; GBDT; gradient boosting decision tree; ET, extremely randomized trees; LR, logistic regression.

The efficacy prediction models of etanercept in JIA, based on 47 pre-administration clinical variables, were generated using machine learning. The process of machine learning could be divided into the following steps: (1) data processing; (2) feature selection; (3) model generation and validation. **Figure 2** shows the flowchart of the whole process. Machine learning techniques were implemented in Python 3 (Python 3.6.5) using the package Scikit-learn (Scikit-learn 0.19.1).

### Data Preprocessing

After finishing the collection of variables, we removed the variables with a missing rate of >30%. Also, to get a higher-quality dataset, the individual missing values of the variable were filled with the average value of the group to which the individual belongs (responders/non-responders). For example, we used the average SJC values of the “responders” group and “non-responders” group to fill the missing value of individuals in the corresponding group respectively.

### Feature Selection

**Figure 3:**
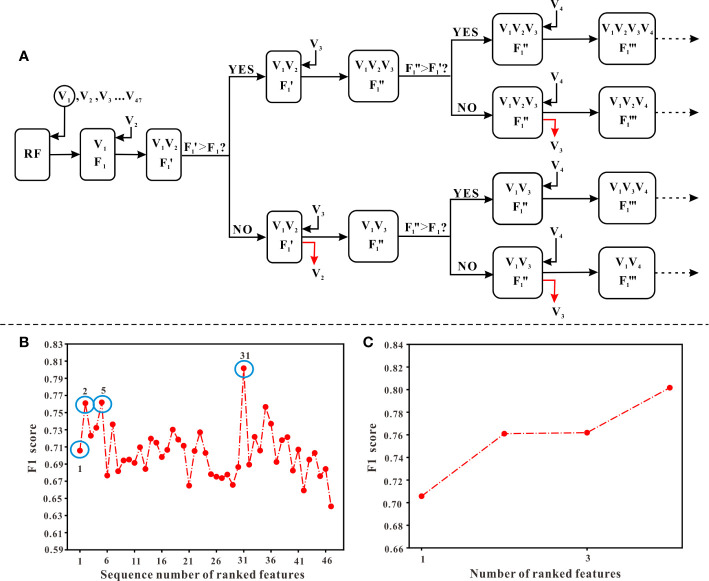
The procedure of feature selection. **(A)** The flowchart of selecting features. Ranked variables according to their information gain values (from high to low, see ). Starting from V_1_ (the first variable) which has the highest information gain values, variables were input into the model generated by RF algorithm in order. A corresponding “F_1_” score, calculated when adding a new variable, should be compared with the previous “F_1_” score. Only when the “F_1_” score was higher than the previous “F_1_” score, the new variable could remain in the model and continued to input the next variable. Otherwise, it should be abandoned and continued to input the next variable. **(B)** The overall variation curve of F_1_ scores of 47 variables. Variables of sequences 1, 2, 5, 31 marked with blue circles were the variables finally selected, including TJC, Time interval, LYM, and Weight. **(C)** The variation curve of F_1_ scores of TJC, Time interval, LYM, and Weight.

In this study, the stepwise forward and backward method based on information gain (IG) was used for feature selection. The IG is defined as the effectiveness of attributes to classify the training data, which is measured by the amount of entropy of the class decreases (Motwani et al., 2017). The process of feature selection was as follows: First, aiming to obtain an optimal feature sequence, we calculated the IG value of each variable, then ranked them according to their IG values from largest to smallest. Next, we added one of the features at a time into the model generated by Random Forest (RF) algorithm and calculated the F1 score of the model (starting from the variable with the largest IG value, until the last variable). Meanwhile, from the start of the second-generated model, which means the model generated by the first two variables with the largest IG values, the F1 score of the new model (F1’) needed to be compared with the previous F1 score derived from the last model. If F1’ is less than F1, the variable added recently should be removed and then added another new variable to generate the model. Otherwise, the variable added recently should be kept in the model and continued to add another new variable. When all variables have completed the above procedure, those variables that met the criteria were the optimal combination of variables, which would be selected to generate the final model. The flowchart of feature selection is shown in **Figure 3A**.

### Model Generation and Validation

Five machine learning algorithms were used to generate predictive models, including Extreme Gradient Boosting (XGBoost), Random Forest (RF), Gradient Boosting Decision Tree (GBDT), Extremely Random Trees (ET) and Logistic Regression (LR). First, the dataset was randomly divided into a training set and a test set according to the ratio of 8:2. Because of the small sample size, we randomized and stratified 87 samples 50 times according to the ratio of 8:2. Fifty different combinations of training and test sets were therefore obtained. Second, the training set was used for model generation, while the test set was used to evaluate the predictive performance of models. This process was repeated 50 times using different training and test sets, and the final performance was obtained by mode calculation over the 50 evaluation values. In this study, a model was defined as the final result of modeling using an algorithm, and the corresponding 50 classifiers were the intermediate results. Therefore, as for each model, the performance indicators such as AUC, accuracy, sensitivity, and specificity were the mode of 50 evaluation values respectively, which followed the concept of majority voting to obtain a more fair and stable effect. In addition, XGBoost, GBDT, and LR algorithms needed combining with the synthetic minority oversampling technique (SMOTE) to deal with data imbalance effectively. Finally, we further collected data from 14 patients for external validation of the above five models. For each model, the final output was obtained by calculating the mode of 50 outputs while input the variables into the 50 classifiers.

## Results

### Patient Characteristics

A total cohort of 87 JIA patients was used for developing models, while 14 patients were further collected to external validate models built by 5 algorithms. Among the 87 patients, the proportion of male patients was 52.9%, and the majority types of JIA patients were polyarthritis and oligoarticular (42.5% and 44.8%), while only 12.7% of patients were other types of JIA. The mean onset age was 5.8 years, and the mean age of etanercept start was 6.2 years. **Table 1** shows the baseline characteristics of 87 patients. According to the DAS44/ESR-3 simplified standard, 58 patients had a good response to etanercept (responders), while 29 were non-responders.

**Table 1:** Baseline characteristics of 87 patients.

Characteristics	Data (n=87)
Gender, n (male/female)	46/41
Age of disease onset, years, (mean ± SD)	5.8 ± 3.0
Age of etanercept start, years, (mean ± SD)	6.2 ± 3.0
Time interval*, months, (mean ± SD)	10.1 ± 13.2
Oligoarticular JIA, n	39
Polyarticular JIA, n	37
Other types JIA**, n	11
Swollen joint count, median (range)	2(0–27)
Tender joint count, median (range)	2(0–32)
CRP, mg/L, (mean ± SD)	39.44 ± 47.32
ESR, mm/h, (mean ± SD)	44.18 ± 33.24
RF-IgG, U/ml, (mean ± SD)	32.72 ± 65.74
Etanercept dose at start, mg, median (range)	12.5(6.25–25.0)

*Time interval, the time from disease onset to initiation of etanercept treatment.

**Other types JIA, included systemic JIA, psoriatic JIA and so on.

### Feature Selection

According to the ranking result of IG values, TJC had the largest IG values while the value of gender was the smallest (see **Figure 1**). In the process of stepwise forward and backward modeling with the RF algorithm, the overall variation curve of F_1_ scores of 47 variables is illustrated in **Figure 3B**. The four circled variables were excavated as the optimal combination, including TJC, Time interval, LYM, and Weight. Their F_1_ scores variation curve is shown in **Figure 3C**.

### Model Performance and Comparison

**Table 2** shows the performance results of 5 prediction models evaluated using the test set, and the results were expressed as mode. Accuracy and AUC of XGBoost are the best of the five, reaching 72.22 and 79.17%, respectively. The sensitivity result of the GBDT algorithm is the best, which is 83.33%. As for the specificity result, the ET algorithm is the best, with a value of 83.33%. These results from the table demonstrate that the XGBoost model has the best predictive performance.

**Table 2:** The results of predictive performance of the models.

Models	Sensitivity (%)	Specificity (%)	Accuracy (%)	AUC (%)
XGBoost	75.00	66.67	72.22	79.17
RF	75.00	66.67	72.22	72.22
GBDT	83.33	50.00	72.22	73.61
ET	58.33	83.33	66.67	73.61
LR	75.00	66.67	66.67	70.83

XGBoost, extreme gradient boosting; RF, random forest; GBDT, gradient boosting decision tree; ET, extremely randomized trees; LR, logistic regression; AUC, area under the curve.

**Figure 4:**
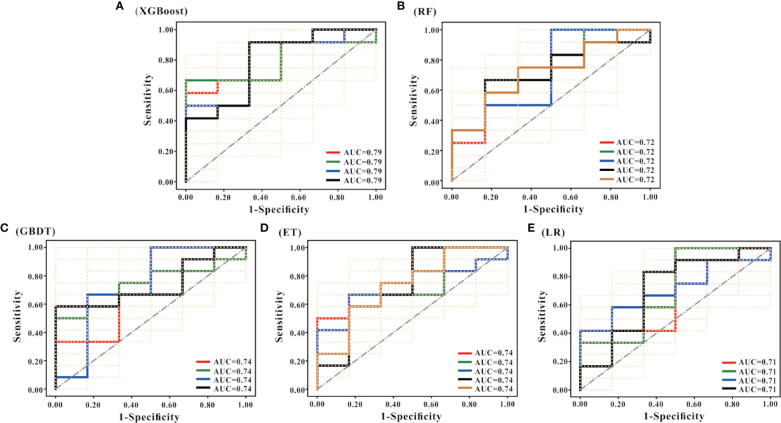
ROC curves of the models. **(A–E)** are the ROC curves of the models generated by the XGBoost, RF, GBDT, ET, and LR algorithms based on the optimal feature subset, respectively. Each algorithm generated models using 50 different training sets. Therefore, each algorithm has 50 ROC curves. As shown in **(A)**, the mode of AUC is 0.79 together with 4 ROCs; the mode of AUC in **(B)** is 0.72, and there are 5 ROCs; the mode of AUC in **(C)** is 0.74, and there are 4 ROCs; the mode of AUC in D is 0.74, with a total of 5 ROCs; and the mode of AUC in € is 0.71, with a total of 4 ROCs. According to the combination of the 50 ROC distributions and AUC values, the XGBoost model has the best prediction performance, and the LR model has the worst prediction performance.

Besides, it can be seen from the ROC curve (**Figures 4A–E**) that the XGBoost model has a ROC curve closest to the upper left corner with the largest AUC (**Figure 4A**), indicating that the model has the best classification performance. Comparing the ROC curve distribution and AUC of 5 models, the classification performance of the LR model is the worst (**Figure 4E**).

Through integrating the results of sensitivity, specificity, accuracy, AUC and ROC curve, the predictive performance of XGBoost model is the best.

### External Verification and Clinical Application

Data from extra 14 patients was used to verify the classification performance of each model. The sensitivity of XGBoost is the best among the five algorithms (81.82%), followed by RF and GBDT (72.73%), ET and LR are the worst (54.55%). As for accuracy, XGBoost still has the best result (64.29%), while the remaining four algorithms (57.14%) are all lower than XGBoost. Thus, the classification performance of XGBoost is the best according to the above results. The results of the mixed matrix were shown in **Figure 1** in supplementary.

We randomly selected 2 of 14 JIA patients and input their TJC, Time interval, LYM, and Weight data into the XGBoost model to predict the response to etanercept. Treatment outcomes were correctly predicted in both patients (see **Table 2 in Supplementary**).

## Discussion

Despite the individual difference of etanercept response in JIA is considerable, there’s still a lack of simple and reliable efficacy predictive model. Therefore, we established a series of EMR-based efficacy prediction models of etanercept in JIA using machine learning. Four important variables were excavated that may influence the response, including TJC, Time interval, LYM, and Weight. The model with the above 4 variables generated by the XGBoost algorithm has the best predictive performance which could accurately identify 79.17% of patients.

To our knowledge, this is the first article using machine learning to build a simple model to predict etanercept response to JIA. We developed and validated a pre-administration machine learning prediction model in detail. At present, several reports have been published to explore the factors influencing the efficacy of etanercept in the treatment of JIA (Otten et al., 2011; Solari et al., 2013; Geikowski et al., 2014; Kearsley-Fleet et al., 2016). For example, Tilman et al., used ACR Pedi 70 (American College of Rheumatology Pediatric Criteria) as the evaluation criteria of etanercept response and built a predictive model using logistic regression (LR) (Geikowski et al., 2014). Childhood Health Assessment Questionnaire (CHAQ), co-treatment with corticosteroids, onset age, and the systemic JIA category were excavated to influence the efficacy of etanercept. However, this study only reported AUC as the indicator of model performance, which was 64.6%, lower than our results (79.17%). While several other studies used traditional logistic regression or Cox regression for modeling (Otten et al., 2011; Solari et al., 2013; Kearsley-Fleet et al., 2016). Some variables such as onset age and JIA category were excavated. However, these studies did not provide specific predictive performance. Because of the unknown efficiency and generalization performance of the models, it is hard to access their extension and application ability.

From the studies discussed above, the predictive performance of those models is not good enough. Part of the reason may be that only the traditional methods like LR were used in modeling, which may not be the best option. In recent years, the rapidly developing machine learning technology has been widely applied in the medical field. This technology can not only predict disease progression, assist diagnosis and evaluate prognosis, but also provide a new method for predicting drug efficacy. Thanks to the powerful computing capacity of computers, the mass medical data can be analyzed, trained and modeled in a short period. Therefore, it is more efficient to explore the correlation between clinical variables and drug efficacy or predict drug efficacy through the trained models. In our previous study, we first established a model to predict the efficacy of methotrexate in JIA using machine learning (Mo et al., 2019). Also, there have already been prediction models generated by multiple machine learning methods for the efficacy of etanercept in psoriasis and ankylosing spondylitis (Liu et al., 2019; Tomalin et al., 2019). Similarly, we used a variety of advanced machine learning algorithms (XGBoost, RF, GBDT, ET) and traditional LR to generate models. XGBoost, RF, GBDT, and ET are all part of ensemble learning. The purpose of ensemble learning is to improve the generalization ability and robustness of a single learner by combining the predicted results of multiple base learners (Dietterich, 2000; Biau, 2012; Chen and Guestrin, 2016; Ke et al., 2017). XGBoost effectively prevents overfitting, but its algorithm parameters are relatively plentiful and complex. The training speed of RF is fast, which can process high-dimensional data sets, and has the ability to deal with unbalanced classification data. But it is easy to overfit classification problems with high noise. GBDT is sensitive to outliers but the training speed is relatively slow. ET is a variant (or extension) of RF. The two are similar, and the variance of ET model is usually smaller than that of RF. LR, on the other hand, is the recognized baseline model (Agresti, 2007). Therefore, combining and comparing these algorithms can present our research results in a more objective and comprehensive manner, which is also conducive to obtaining the best model. To our study, XGBoost model had the strongest prediction performance, while LR model was the weakest. The AUC and accuracy of LR were significantly lower than the other four machine learning methods (see **Table 2**). Our results are similar to those relevant studies, indicating that the machine learning methods have better predictive performance than the traditional statistical methods. This may be due to the limitation of over-fitting and multicollinearity, which cause LR processing mass variables weakly (Lee et al., 2018).

Additionally, we used the stepwise forward and backward method based on information gain to select features and found the optimal combination of feature subsets, which effectively solved the curse of dimensionality problem due to the small sample size in this study (see **Figure 3**). Four optimal features were excavated, including TJC, Time interval, LYM, and Weight. Compared to other studies (Liu et al., 2019; Tomalin et al., 2019), these features were completely derived from routine monitoring and more suitable for clinical application. The model built by Lewis et al. might not reflect clinical reality because the data used for modeling were derived from phase III clinical trial which had strict inclusion criteria (Grapow et al., 2006). The other study (Liu et al., 2019) focused on IgG galactosylation status and gene influence on efficacy, which required additional expensive detection. Therefore, it’s not conducive to clinical application. TJC refers to the number of joints pain at rest with pressure (Scott and Scott, 2014), which reflects the disease activity of rheumatoid arthritis (RA) and JIA (Smolen et al., 1995; Riazzoli et al., 2010). According to several reports that explored the correlation between etanercept efficacy and JIA category, etanercept had poor efficacy in systemic JIA (Otten et al., 2011; Geikowski et al., 2014). This is consistent with our result. Time interval is the time from disease onset to initiation of etanercept treatment. Previous studies found that the earlier use of drugs, the more easily disease became inactive within 6 months, especially for biological agents. Compared with other disease modifying antirheumatic drugs, using biological agents earlier can get a greater improvement (Tynjälä et al., 2011; Wallace et al., 2012). This suggests that clinicians should use etanercept as soon as possible to maximize the efficacy, which is consistent with our findings. Lymphocytes play an important role in JIA. Studies have shown that the pathogenesis of RA and JIA may be related to the apoptosis inhibition of lymphocytes in synovial fluid and the persistent infiltration of T cells in rheumatoid synovium (Murray et al., 1996; Smolewska et al., 2006). Additionally, activated memory B cells can be antigen-presenting cells in JIA and participate in inflammatory responses. The effect of etanercept on B cells was achieved by reducing the B cell-activating factor in serum and increasing Tfh cells (Morbach et al., 2011; Glaesener et al., 2014). TNF is an important cytokine secreted by lymphocytes (T cells, B cells, etc.). The mechanism of etanercept in JIA except competitively inhibits TNF binding to its receptor and exerts anti-inflammatory effects, but also reduces the proportion of Th1 lymphocytes secreting TNF in the peripheral blood (Maggi et al., 2014). Thus, lymphocytes are closely related to the pathogenesis of JIA and the mechanism of etanercept. The above findings were validated by our results that lymphocytes contributed to the treatment outcomes of etanercept. Except for energy storage tissue, adipose tissue can also secrete adipocytokines, which resulted in immunomodulation and mediated inflammatory (Tilg and Moschen, 2006). The occurrence of metabolic or inflammatory diseases (such as RA) is usually associated with abnormally elevated adipocytokine in plasma (Muller-Ladner and Neumann, 2009). Studies have also shown that RA patients with a high BMI had a poor response to anti-TNF biological agents (Klaasen et al., 2011; Gremese et al., 2014; Ottaviani et al., 2015). And adipose cytokine levels and BMI are closely related to body weight. Similar to the above studies (Muller-Ladner and Neumann, 2009; Klaasen et al., 2011; Gremese et al., 2014; Ottaviani et al., 2015), we excavated bodyweight that could influence etanercept response in JIA.

In general, this well-performing model can be easily applied to predict the short-term efficacy of etanercept in the treatment of JIA (see **Table 2** and **Figure 1 in Supplementary**). Due to few patients of external validation, those non-responders could not be well validated temporarily. Additionally, some studies found that mixture modeling with pre- and post-administration variables could significantly improve the predictive performance of the model (Mo et al., 2019; Tomalin et al., 2019). Therefore, we intend to further generate models with variables after administration. The retrospective studies cause the lack of evaluation data like CHAQ, conducting prospective studies for modeling and validation is also a method to improve the predictive performance of models.

We used advanced machine learning algorithms for the first time to generate a pre-administration model with good prediction performance for the efficacy of etanercept in JIA. The variables excavated by the model were TJC, Time interval, LYM, and Weight, which are closely related to the disease onset and the mechanism of etanercept. Clinicians and pharmacists can predict the response to etanercept of JIA patients through this simple and accurate model before administration, to avoid treatment failure or adverse effects caused by experimental exploration.

## Data Availability Statement

The raw data supporting the conclusions of this article will be made available by the authors, without undue reservation.

## Ethics Statement

This study was reviewed and approved by the ethics committee of Guangzhou women and children’s medical center (no. 2016021645) and conducted according to the Helsinki declaration. Written informed consent from the participants’ legal guardian/next of kin was not required to participate in this study in accordance with the national legislation and the institutional requirements.

## Author Contributions

Conceptualization, XM, YC, HLia, and HZ. Data curation, XM, SZ, HLi GL, GS, FH, YX, and PZ. Formal analysis, XC, CI, GL, and HLia. Funding acquisition, XM, YC, and HZ. Investigation, XM, GS, YH, and FH. Methodology, XM, XC, JL, and HLia. Project administration, XM, YC, HLia, and HZ. Resources, YC, HLia, and HZ. Software, XC and CI. Supervision, XM. Validation, GS, YX, and PZ. Writing – original draft, XM, XC, and CI. Writing –review and editing, XM, XC, CI, YC, HLia, and HZ.

## Funding

This research was supported by grants from the National Natural Science Foundation of China (grant no. 81603203), Health Commission of Guangdong Province (grant no. A2016400), Guangdong Pharmaceutical Association Program (grant no. 2015FS10 and 2015SW05), Guangzhou Institute of Pediatrics/Guangzhou Women and Children’s Medical Center (grant no. YIP-2018-020).

## Conflict of Interest

The authors declare that the research was conducted in the absence of any commercial or financial relationships that could be construed as a potential confict of interest.
