# Supplementary material for: Early Prediction of Clinical Response to Etanercept Treatment in Juvenile Idiopathic Arthritis Using Machine Learning
Source: Front Pharmacol. 2020 Jul 31;11:1164. doi: 10.3389/fphar.2020.01164 (PMC7411125; doi:10.3389/fphar.2020.01164)
Supplement: Supplementary file 1 [file DataSheet_1.docx]

**SUPPLEMENTARY**

**TABLES**

**Table 1 The full name and abbreviation of variables**

| **Full name of variables** | **Abbreviation name** | **Full name of variables** | **Abbreviation name** |
| --- | --- | --- | --- |
| Age of etanercept start | Age of start | Indirect bilirubin | IBIL |
| Age onset | Age onset | Immune globulin A | IgA |
| Albumin | ALB | Immune globulin E | IgE |
| Anti-cyclic citrullinated peptide | Anti-CCP | Immune globulin G | IgG |
| Active partial thrombin time | APTT | Immune globulin M | IgM |
| Aspartate aminotransferase | AST | JIA subtype | JIA subtype |
| Complement 3 | C3 | Lymphocyte | LYM |
| Complement 4 | C4 | Neutrophil | NEUT |
| CD16+CD56+ | CD16+CD56+ | Platelet | PLT |
| CD19+ | CD19+ | Prothrombin time | PT |
| CD3+Abs | CD3+Abs | Red blood cell | RBC |
| CD3+CD4+ | CD3+CD4+ | Rheumatoid factor-IgG | RF-IgG |
| CD3+CD8+ | CD3+CD8+ | Serum creatinine | SCr |
| C-reactive protein | CRP | Swollen joint count | SJC |
| Direct bilirubin | DBIL | Total bilirubin | TBIL |
| The first dose of etanercept on the start | Dose0 | Helper T cells/ Suppressor T cells | Th/Ts |
| Erythrocyte sedimentation rate | ESR | Time interval | Time interval |
| Ferritin | FER | Tender joint count | TJC |
| Fibrinogen | FIB | Thrombin time | TT |
| Gender | Gender | Urea | Urea |
| Blood glucose | GLU | White blood cell | WBC |
| Hematocrit | HCT | Weight | Weight |
| Hemoglobin | HGB | 25-hydroxy-vitamin D | 25-OH-vitD |

CD, Cluster of differentiation cell; CD3+Abs, the absolute value of T cell with Cluster of differentiation 3; CD3+CD4+, the ratio of CD4+ divided by CD3+.

**Table 2 The results of etanercept response of clinical patients predicted by XGBoost model**

| XGBoost  model | Patient  name | Input variables | | | | Output |
| --- | --- | --- | --- | --- | --- | --- |
|  |  | TJC  （n） | Time interval  （m） | LYM  （%） | Weight  （kg） |  |
|  | XXX | 12 | 15.39 | 52 | 12.50 | GR |
|  | YYY | 2 | 4.34 | 37 | 12.30 | GR |

The patients named XXX and YYY in this table were both responded to etanercept in clinic practice. We input their variables from clinical determination into the XGBoost model. Outcomes of both two patients were predicted correctly. Output represents the prediction results of etanercept response.

**FIGURE LEGENDS**

**Supplementary Figure 1.** The mixed matrix results of 5 pre-administration models of the external validation set. For example, when the true labels were GR (good response) and predicted labels were also GR, it revealed that the number of patients with GR was correctly predicted. However, when true labels were GR, while predicted labels were NR (non-response), it revealed that the number of patients with GR incorrectly predicted to NR. As the figure shows, the predicted values of XGBoost model is the closest to the real values, indicating the best performance of prediction. Furthermore, the numbers in the yellow grids represent the number of patients that were accurately predicted.
