# Supplementary figures and images for: Early Prediction of Clinical Response to Etanercept Treatment in Juvenile Idiopathic Arthritis Using Machine Learning
Source: Front Pharmacol. 2020 Jul 31;11:1164. doi: 10.3389/fphar.2020.01164 (PMC7411125; doi:10.3389/fphar.2020.01164)

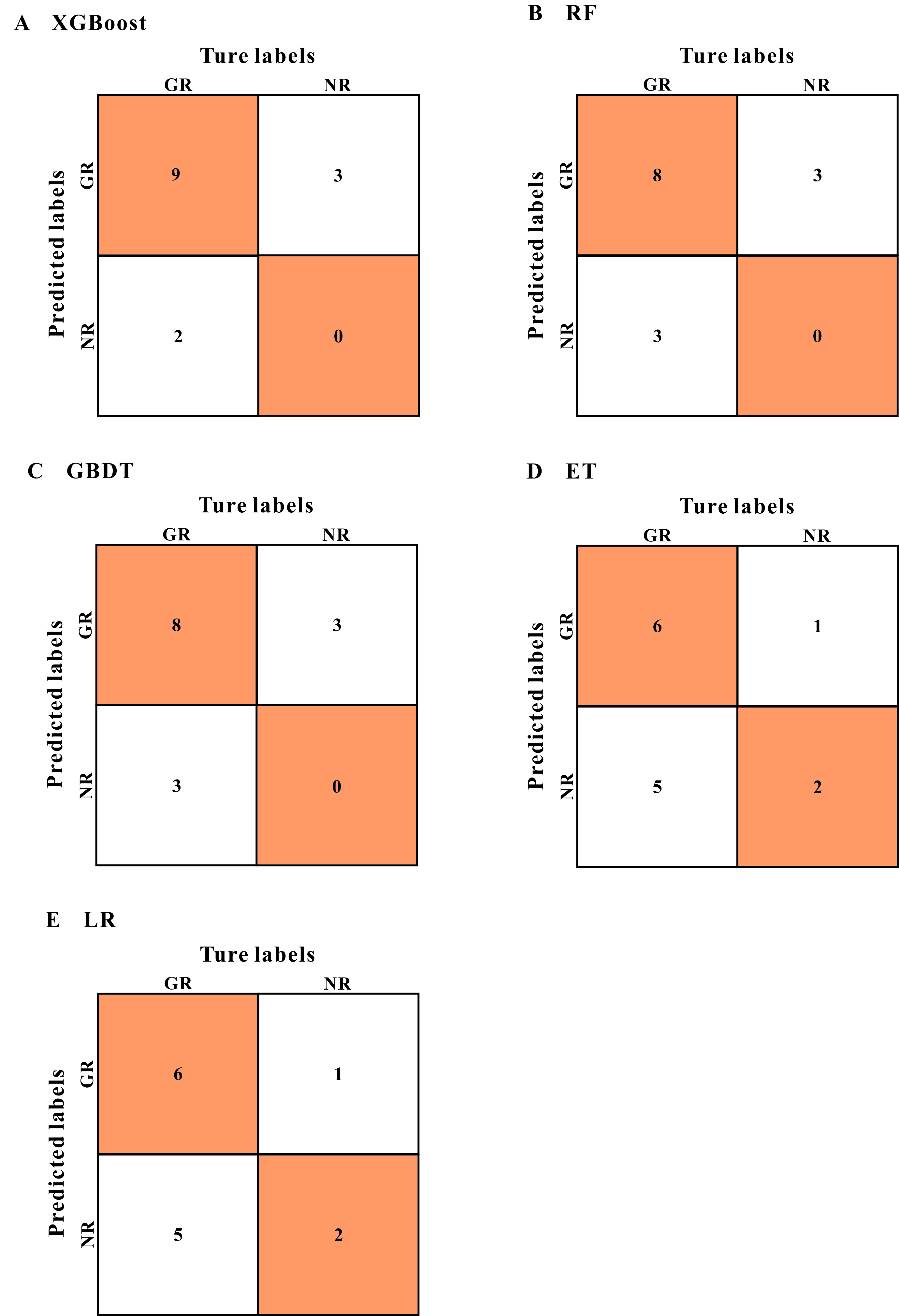

Supplement: Supplementary file 2 [file Image_1.tif]
